# Successful Aging of Korean Older Adults Engaging in Sports Regularly: Health Beliefs, Exercise Adherence Intention, and Health Promotion Behavior

**DOI:** 10.3390/healthcare12090890

**Published:** 2024-04-25

**Authors:** Song-Eun Kim, Jun-Su Bae, Eun-Chul Seo, Wi-Young So, Young-Kyun Sim

**Affiliations:** 1 Department of Social Physical Education, Soonchunhyang University, Soonchunhyang-ro, Asansi 31538, Republic of Korea; 20190412@sch.ac.kr; 2 Institution of Sport Contents, Andong National University, 1375 Gyeongdong-ro, Anndong-si 36729, Republic of Korea; junsubae@anu.ac.kr; 3 Department of Physical Education, Wonkwang University, 460 Iksan-daero, Iksan-si 54538, Republic of Korea; eunchulseo17@wku.ac.kr; 4 Sports Medicine Major, College of Humanities and Arts, Korea National University of Transportation, Chungju 27469, Republic of Korea; 5 Sports Welfare Convergence Research Institute, Woosuk University, 443 Samnye-ro, Wanju-gun 55338, Republic of Korea

**Keywords:** exercise adherence, older adults, health beliefs, health promotion behavior, sports, successful aging

## Abstract

The population of older adults is increasing more rapidly in Korea than in any other country, making successful aging a salient need in Korean society. For successful aging, older adults must engage in sports activities regularly. This study determined the relationships among health beliefs, exercise adherence intention, health promotion behavior, and successful aging among older adults who engage in sports activities regularly. The participants were 287 adults aged 65 years or older who live in Korea and exercise regularly. Data were collected through a survey and analyzed using descriptive statistics, correlation analysis, and structural equation modeling. The integrated model lacked explanatory power in terms of goodness of fit, but the alternative model had sufficient explanatory power. The alternative model showed that health beliefs, exercise adherence intention, and health promotion behavior are significantly related and that health promotion behavior positively predicts successful aging. It also showed that health beliefs affect successful aging through the mediation of exercise adherence intention and health promotion behavior. This study is meaningful because it verifies the structural and theoretical relationships among health beliefs, exercise adherence intention, health promotion behavior, and successful aging. As a result, it provides information that can improve the welfare of older adults in Korean society.

## 1. Introduction

Korea has the world’s lowest birth rate and has become a “super-aging society”. According to a survey [1], there were approximately 9.7 million individuals aged 65 years or older in Korea in 2023, which is a sharp increase of 2.7 million from 2017. This population accounted for approximately 18.95% of the country’s total population in 2023 [1]. Experts have predicted that the older adult population in Korea will be approximately 20% of the country’s population in 2025 and will exceed 40% by 2060 [2]. The increase in this population affects all areas of the country and society. Along with the psychological crisis that comes with aging, it induces feelings of alienation; loss of income; changes in relationships with family, friends, and neighbors; health problems; and loss of social status and roles [3].

With the onset of becoming a super-aging society, a question that has arisen in Korean society is “How will we live happily in old age?” Researchers have been trying to find an answer to this question for a long time and have defined this concept as “successful aging” [4,5]. This concept has been discussed since ancient times, but it gained the attention of many researchers when Rowe and Kahn [6] compared “usual aging” and “successful aging”. Since then, researchers such as Ryff [7] and Baltes and Baltes [8] have contributed to this research by proposing theories on successful aging. Rowe and Kahn [6] presented a social participation model that showed that one can have a successful old age through active and continuous participation in life. In addition, this model showed that successful aging has three main characteristics: low risk of disability due to disease, high cognitive and physical function, and active participation in life, particularly in social relationships. Meanwhile, Estebsari et al. [9] showed that successful aging can be considered a multidimensional concept that has physical, social, and psycho-emotional aspects.

Numerous empirical studies have demonstrated that engaging in health promotion behaviors plays a pivotal role in facilitating successful aging. Engaging in health promotion behaviors involves adopting a positive approach to life and strategies aimed at enhancing well-being and self-realization [6,7,8,9,10]. Such behaviors not only help to prevent illnesses and reduce morbidity rates but also contribute to improving the overall quality of life while reducing healthcare costs. Chou et al. [11] conducted a meta-analysis to analyze the effects of exercise (a health promotion behavior) on physical function, the performance of activities of daily living, and the quality of life among frail older adults. They found that exercise increases walking speed and improves the balance and daily life of frail older adults. It also helps to improve performance in motion. Liberman et al. [12] conducted a systematic review to analyze the effects of exercise on muscle strength, body composition, body function, and inflammation among older adults and found that exercise has a moderate effect. Menec [13] conducted a six-year longitudinal study to analyze the relationship between daily physical activity and successful aging. The results showed that the overall activity level is positively related to happiness in older people and is associated with better function and reduced mortality six years later [13]. Furthermore, numerous studies have directly or indirectly shown the effectiveness of regular sports and exercise activities for successful aging [14,15,16].

Although participating in regular sports and exercise activities [17] facilitates successful aging, engaging in these activities is not as easy as expected for older people. There are many obstacles to older adults’ participation in sports and exercise, such as one’s personality, the deterioration of physical health, financial problems, psychological changes, and relationships with the people around oneself. While these factors may hinder exercise participation, they may also promote it [18]. Researchers have made various attempts to understand the process through which older people participate in exercises that promote successful aging. In the studies known to date, an individual’s decision to participate in exercise is made through various cognitive processes, one of which is intention [18]. In other words, one’s intention to participate in exercise influences the actual exercise participation or continued behavior [19].

One’s intention to participate in or continue exercising comes from one’s beliefs, and the theory that explains this phenomenon is the Health Belief Model (HBM) [20]. The HBM explains how one’s beliefs about health induce health behaviors (such as participation in exercise) [21]. The key variables in the HBM are awareness of disease susceptibility, awareness of disease severity, awareness of benefits, and awareness of obstacles. First, awareness of disease susceptibility refers to the awareness that health problems may occur if one does not exercise. Older adults who exercise regularly [17] may be concerned that their health will deteriorate if they stop exercising. Second, awareness of disease severity refers to the awareness that, if one contracts a disease, it will hamper their quality of life. It includes an awareness of the serious medical consequences (such as death, disability, and extreme pain) and personal consequences (such as interruption of work and endangerment of one’s home) of contracting a disease. Third, awareness of benefits refers to the awareness that exercise benefits one’s health. To adopt a health behavior (such as participation in exercise), one must recognize the benefits of exercise, even if one is aware of disease susceptibility and disease severity. If one does not recognize that exercise helps to prevent diseases, one will not participate in the exercise, even if they are aware of the possibility and severity of the disease. Finally, awareness of obstacles refers to the awareness of factors that hinder exercise participation. Even if one is aware of the benefits of exercise for disease prevention, one considers its limitations. More specifically, one performs a “cost–benefit” analysis of the benefits and limitations of participation in exercise. For example, an older individual may be aware of the fact that participating in exercise is good for them, but they may be hesitant because of the financial burden associated with exercise participation [20,21].

In the field of exercise science, many efforts have been made to explain the exercise behavior of older people using the HBM [22,23]. For example, Yu et al. [24] surveyed 329 older adults living in China and found that the higher their health beliefs, the higher their participation in physical activities and life satisfaction. Harrison et al. [25] used the HBM to gain a broader understanding of the barriers to, motivations for, and benefits of physical activity and exercise participation among older adults living in Washington, DC, USA. These studies have shown that the HBM is an effective model for predicting exercise participation and happiness among older adults. However, despite the HBM’s theoretical explanatory power, research applying the model among Korean older adults is rare. There is a need to confirm whether the HBM has sufficient explanatory power in the context of Korea. In addition, most studies have used the HBM to confirm its explanatory power in predicting exercise activities among older adults, but there is still a lack of evidence of its ability to predict successful aging. This study aimed to analyze the relationship among health beliefs, exercise adherence intention, health promotion behavior, and successful aging among older adults who participate in exercise and sports regularly. Based on empirical evidence of the importance of exercise for successful aging and the HBM for predicting exercise participation [14,24], this study hypothesized that the HBM positively predicts successful aging. In other words, it was assumed that health beliefs, exercise adherence intention, health promotion behavior, and successful aging would be positively related to each other. This endeavor may expand our understanding of the process of exercise participation and help to promote successful aging among Korean older adults.

## 2. Materials and Methods

### 2.1. Participants and Data Collection

The participants in the study were 287 individuals aged 65 years or older in Korea who regularly participate in sports. Regular participation was defined as participation at least three times a week for six months [25]. The participants were recruited from September to December 2023 using purposive sampling. First, the researchers contacted the officials of recreational sports organizations where older people participated (for instance, clubs and sports centers) and asked for their cooperation in the study. A notice to recruit research participants was posted at the collaborating institutions, and individuals who wanted to participate were recruited as participants. The research team visited the institutions on the date posted in the notice and surveyed the participants. The researchers visited a total of 13 institutions, and surveys were conducted on different days at each location. At each institution, the researchers distributed the questionnaire to the participants after clearly explaining the purpose and methods of the study. The participants filled the questionnaire themselves, and filled-in questionnaires were collected immediately by the researchers.

To deal with the ethical issues that may arise during the study, the researchers explained the purpose of the study to the participants and made efforts to obtain their voluntary consent. Given that the participants were older adults, the researchers explained the use and processing of their data in greater detail. Additionally, if a participant wished to stop participating in the study, their data were excluded from the analysis and destroyed. If they did not want their data to be used in the study, they could ask to be excluded from the study even after the completion of the questionnaire. This study was approved by the Institutional Review Board of Yong In University (IRB No. 2-1040966-AB-N-01-2311-HR-332-1) and conducted in accordance with the Declaration of Helsinki. In addition, all participants provided a written informed consent.

### 2.2. Instruments

The measurement tool to achieve the purpose of this study was a structured questionnaire, and all variables and questions were constructed based on previous research consistent with the purpose of this study. The questionnaire contained 81 questions: 4 questions about participants’ sociodemographic characteristics, 26 questions about health beliefs, 5 questions about exercise adherence intention, 15 questions about health promotion behavior, and 31 questions about successful aging. All questions were answered on a 5-point Likert scale.

#### 2.2.1. Health Beliefs

Health beliefs were measured using the Korean version of the sports health belief questionnaire [26], which was developed based on the model proposed by Janz and Becker [27]. This questionnaire measures the main factors affecting one’s health beliefs about exercise participation. The questionnaire consists of 26 questions across six factors: 5 questions about psychological benefits, 4 questions about physical benefits, 4 questions about social benefits, 5 questions about perceived barriers, 4 questions about susceptibility, and 4 questions about severity. Higher scores indicate more favorable health beliefs.

#### 2.2.2. Exercise Adherence Intention

Exercise adherence intention was measured using the Exercise Adherence Rating Scale developed by Newman-Beinart et al. [28]. This scale consists of a single factor that is measured using six questions on exercise participation. Higher scores on the scale indicate higher levels of exercise adherence intention.

#### 2.2.3. Health Promotion Behavior

Health promotion behavior was measured using the Korean version of the questionnaire [29] developed by Walker et al. [30]. The questionnaire consists of 15 questions across three factors: 5 questions on health responsibility, 5 questions on interpersonal support, and 5 questions on exercise. Higher scores on this questionnaire indicate higher levels of health promotion behavior.

#### 2.2.4. Successful Aging

Successful aging was measured using the Korean Elderly’s Successful Aging Scale [31], which was developed based on Ryff’s theory [7]. This scale comprises 31 questions across six factors: 9 questions on autonomous life, 6 questions on self-completion orientation, 5 questions on positive life participation, 5 questions on satisfaction with one’s offspring, 3 questions on self-acceptance, and 3 questions on the acceptance of others. Higher scores on this scale indicate a greater degree of successful aging.

### 2.3. Data Analysis

All statistical analyses were performed using SPSS and AMOS (version 24.0; IBM Corp., Armonk, NY, USA). First, descriptive statistics were calculated from frequency analysis and normality tests. They were calculated using means, standard deviations, and skewness and kurtosis values. Skewness and kurtosis were calculated to verify normality. When the skewness value is 3.00 or less and kurtosis value is 8.00 or less, the data collected can be judged to be normal [32]. Second, a confirmatory factor analysis (CFA) was performed using the maximum likelihood estimation method, in addition to a reliability analysis using Cronbach’s α, to determine the validity and reliability of the measurement tools used. The suitability of the CFA model was judged through *χ*^2^, comparative fit index (CFI; >0.900), Tucker–Lewis index (TLI; >0.900), standardized root-mean-squared residual (SRMR; <0.080), and the root-mean-square error of approximation (RMSEA; <0.080) [32]. Third, a bivariate correlation analysis was conducted using Pearson’s product-moment correlation coefficient. The closer the coefficient is to 1.000, the higher the correlation. Fourth, a structural equation model analysis was conducted to identify the relationship among health beliefs, exercise adherence intention, health promotion behavior, and successful aging among older adults who participate in sports activities regularly. The model fit is considered very good when the CFI > 0.900, TLI > 0.900, SRMR < 0.080, and RMSEA < 0.080. Before conducting the structural equation model analysis, the validity of the factor structure was examined and the suitability of the measurement model was reviewed using Anderson and Gerbing’s [33] two-step approach. Finally, an analysis was conducted using phantom variables to determine indirect effects in the path from health beliefs to successful aging. Phantom variables are useful for verifying the significance of the mediating effect of each variable when there are two or more mediating variables [34]. Bootstrapping was performed 2000 times, and the confidence interval estimation was set to the 95% confidence interval level of the bias-corrected method. If zero was not included in the confidence interval, the mediating effect was considered to be significant. Statistical significance was set at *p* < 0.05.

## 3. Results

### 3.1. Characteristics of the Participants

Table 1 shows the characteristics of the participants. Among the participants, most were male (62.72%). On average, the participants were aged 70.01 years (standard deviation = 4.43, range = 65–91 years) and participated in exercise for 4.61 years (standard deviation = 1.74). They participated in exercise an average of 3.31 times a week, and they exercised for approximately 1.89 h per session. Furthermore, they participated in hiking, park golf, swimming, badminton, gate ball, golf, dance, cycling, futsal, tennis, soccer, and table tennis. Nearly half of the participants (49.83%) lived from their earned income. Most participants lived with their spouse (59.23%).

### 3.2. Validity and Reliability of the Measurement Tools

Table 2 presents the results of analyzing the validity and reliability of the measurement tool of each variable. In analyzing the validity and reliability of the measurement tool for health beliefs, the loading value of one question on severity (“I do not think that my health will worsen due to a lack of exercise”) was not significant. Therefore, the question was removed. After removing it, the model fit indices were *x*^2^ = 494.293, *df* = 258 (*p* < 0.001), CFI = 0.919, TLI = 0.906, SRMR = 0.057, and RMSEA = 0.057 (95% confidence interval (CI) = 0.049~0.064). Reliability was confirmed, as the Cronbach’s α values were 0.880, 0.816, 0.809, 0.860, 0.922, and 0.868 for psychological benefits, physical benefits, social benefits, perceived barriers, susceptibility, and severity, respectively.

When analyzing the validity and reliability of the measurement tool for exercise adherence intention, the model fit indices were *x*^2^ = 16.690, *df* = 8 (*p* < 0.001), CFI = 0.991, TLI = 0.982, SRMR = 0.020, and RMSEA = 0.062 (95% CI = 0.016–0.103). Reliability was confirmed, as the Cronbach’s α was 0.894. In analyzing the validity and reliability of the measurement tool for health promotion behavior, the model fit indices were *x*^2^ = 234.106, *df* = 84 (*p* < 0.001), CFI = 0.923, TLI = 0.904, SRMR = 0.070, and RMSEA = 0.079 (95% CI = 0.067–0.091). Reliability was confirmed, as the Cronbach’s α values were 0.810, 0.83, and 0.886 for health responsibility, interpersonal support, and exercise, respectively.

When analyzing the validity and reliability of the measurement tool for successful aging, the loading value of one question on self-completion orientation (“I donate materials to others when I get the opportunity”) was not significant. Therefore, the question was removed. After removing it, the model fit indices were *x*^2^ = 787.442, *df* = 379 (*p* < 0.001), CFI = 0.914, TLI = 0.901, SRMR = 0.055, and RMSEA = 0.061 (95% CI = 0.055–0.067). Reliability was confirmed, as the Cronbach’s α values were 0.807, 0.821, 0.912, 0.886, 0.842, and 0.822 for autonomous life, self-completion orientation, positive life participation, satisfaction with one’s offspring, self-acceptance, and acceptance of others, respectively.

### 3.3. Normality of the Data Collected and Correlation among the Factors

Table 3 shows the means, standard deviations, skewness and kurtosis values, and correlation coefficients of the variables. The mean of the variables ranged from 3.602 to 4.337. The standard deviations ranged from 0.438 to 0.754. The skewness values ranged from −1.581 to 0.114, while kurtosis values ranged from −0.650 to 4.917. According to Kline [32], the data can be judged to be normal when the skewness value is three or less and kurtosis value is eight or less. Although the skewness and kurtosis values of the variables were slightly high, they met the normality standards. The results of the bivariate correlation analysis show that the variables have partially significant correlations. The correlation between exercise adherence intention and exercise was the highest (r = 0.593; *p* < 0.01), whereas the correlation between susceptibility and exercise adherence intention and that between severity and autonomous life were the lowest (r = 0.118; *p* < 0.05).

### 3.4. Structural Equation Model

#### 3.4.1. Model 1: Integrated Model

**Figure 1:**
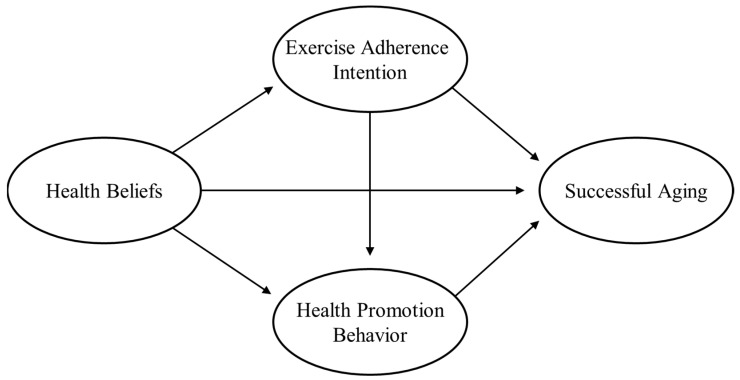
Integrated model.

The integrated model (multiple mediation model), as shown in Figure 1, was used to investigate the relationship among health beliefs, exercise adherence intention, health promotion behavior, and successful aging among Korean older adults who participate in sports regularly. The goodness of fit indices of the model were *x*^2^/*df* = 4.428 (*x*^2^ = 810.241, *df* = 183, *p* < 0.001), CFI = 0.787, TLI = 0.756, SRMR = 0.149, and RMSEA = 0.109 (95% CI = 0.102–0.117). Even if the goodness of fit index criteria were to be interpreted leniently, this model was difficult to accept. This was because, even if the path coefficients in this model showed statistically significant values, the model fit indices did not meet the acceptance criteria. Therefore, an alternative model was needed. The path from health beliefs to successful aging and the path from exercise adherence intention to successful aging were removed to create an alternative model.

#### 3.4.2. Measurement Model Verification

Anderson and Gerbing [33] recommend verifying the measurement model before verifying the structural model. Therefore, the researchers first reviewed the suitability of the measurement model after saturating the path verified in the structural model. The goodness of fit indices of the model were *x*^2^/*df* = 2.108 (*x*^2^ = 204.504, *df* = 97, *p* < 0.001), CFI = 0.947, TLI = 0.934, SRMR = 0.060, and RMSEA = 0.062 (95% CI = 0.050–0.074), with the fit indices meeting the critical values [32]. Additionally, as shown in Table 4, the standardized coefficient of each latent variable explaining the measured variable was greater than 0.509. Thus, the explanatory power of the measured variable also appeared to be satisfactory. Since the measurement model was found to be excellent, the structural model was verified [33].

#### 3.4.3. Model 2: Alternative Model

The goodness of fit indices of the alternative model were *x*^2^/*df* = 2.451 (*x*^2^ = 240.214, *df* = 98, *p* < 0.001), CFI = 0.930, TLI = 0.914, SRMR = 0.082, and RMSEA = 0.071 (95% CI = 0.060–0.083). These fit indices significantly improved compared to the indices of Model 1 (integrated model), and except for SRMR, the fit indices met the criteria for a “good fit”. The SRMR was slightly higher than the standard but still at a sufficiently acceptable level. Therefore, the alternative model was judged to be appropriate.

**Figure 2:**
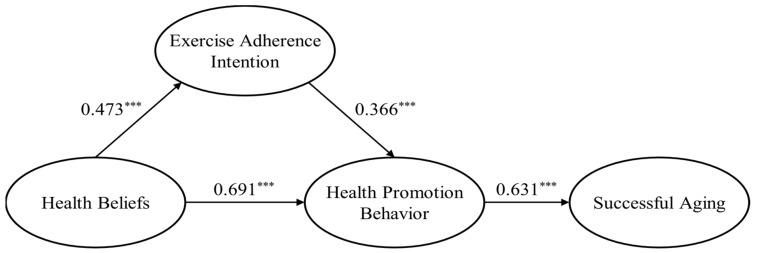
Standardized estimates in the alternative model (*** *p* < 0.001).

Table 5 presents the results of analyzing the direct paths of the alternative model. All direct paths were found to be statistically significant (*p* < 0.001). The effect of health beliefs on exercise adherence intention was *β* = 0.473 (*t* = 3.242). The effect of exercise adherence intention on health promotion behavior was *β* = 0.366 (*t* = 4.095). The effect of health beliefs on health promotion behavior was *β* = 0.691 (*t* = 3.305). The impact of health promotion behavior on successful aging was *β* = 0.631 (*t* = 7.036). Figure 2 presents the standardized estimates of the paths in the research model.

#### 3.4.4. Model 3: Indirect Effect (Phantom Variable) Model

The indirect paths in the research model were analyzed using phantom variables and bootstrapping. Table 6 presents the results of analyzing the indirect paths. All three indirect paths were found to be statistically significant. The effect of health beliefs on health promotion behavior through the mediation of exercise adherence intention was B = 0.301 (*p* < 0.05). The effect of health beliefs on successful aging through the mediation of health promotion behavior was B = 0.770 (*p* < 0.01). The effect of health beliefs on successful aging through the mediation of exercise adherence intention and health promotion behavior was B = 0.193 (*p* < 0.01).

## 4. Discussion

The HBM is one of the most widely used theories to explain exercise behavior, but its application to Korean culture has been very limited. Moreover, studies that have verified models comprising successful aging are difficult to find. This study analyzed the relationships among health beliefs, exercise adherence intention, health promotion behavior, and successful aging among Korean older adults who exercise regularly. The integrated model included the paths from health beliefs and exercise adherence intention to successful aging, but its explanatory power was insufficient. Consequently, an alternative model was created, and its fit was found to be acceptable. In structural equation modeling, researchers can propose alternative models that have a higher theoretical explanatory power or those that provide clearer explanations for the data [35]. The results show that the participants’ health beliefs directly affect their exercise adherence intention and health promotion behavior. Their exercise adherence intention directly affected their health promotion behavior, which positively predicted successful aging. The results also show that health beliefs positively affect successful aging through the mediation of exercise adherence intention and health promotion behavior. The results of this study are similar to those of previous studies that analyzed exercise participation and healthy lives of older adults using the HBM. In particular, Yu et al. [24] conducted a study in a similar Asian culture and found that older people’s health beliefs are closely related to physical activity participation and subjective life satisfaction, supporting the results of this study. The results of this study also support those of studies that demonstrated the relationship among exercise participation, health-promoting behaviors, and successful aging [36,37,38].

The two models analyzed in this study (the integrated and alternative models) provide meaningful information about the successful aging of older adults who exercise regularly. This study found that older adults’ health beliefs and exercise adherence intention do not directly predict successful aging. As it can be seen from the alternative model, health beliefs, exercise adherence intention, and successful aging are explained by the health promotion behavior variable. Interpreting the model of this study, the concerns of older people who exercise, or their perceptions of the benefits of and obstacles to exercise (i.e., health beliefs), are not directly related to the factors that determine a happy life in old age (i.e., successful aging). Exercise adherence intention also did not directly predict successful aging. Instead, health beliefs and exercise adherence intention promoted successful aging through health-improving actions. In other words, beliefs and intentions about health can be related to successful aging when followed by specific actions. In this way, the alternative model in this study, similar to the HBM proposed by Becker and Maiman [20], shows a sequential relationship in which one’s beliefs form intentions, intentions influence behavior, and one’s health behaviors ultimately influence one’s quality of life.

Looking more specifically, the results of this study show that health beliefs influence exercise adherence intention. This relationship is explained in detail by Yardley et al. [39]. They analyzed the attitudes and beliefs of older adults and their intention to participate in strength and balance training (SBT), focusing on the fact that, although SBT programs are effective in preventing falls among older adults, older adults are reluctant to participate in these programs. They analyzed the data of 558 older adults aged 60–95 years and found that the intention to participate in SBT is closely related to the factors related to their attitudes and beliefs. That is, older adults’ concerns about whether SBT exercise would be harmful, tiring, or painful and whether they would be able to perform it positively predicted their intention to participate in SBT. A study targeting older adults in Taiwan [40] also obtained similar results. Therefore, it can be inferred that older adults’ concerns and considerations about their health and their awareness of the benefits of and obstacles to exercise participation (health beliefs) boost their intention to continue exercising.

The results of this study also show that exercise adherence intention positively affects health promotion behavior, while health beliefs directly affect health promotion behavior. These results indicate that the HBM has sufficient explanatory power, even when it is applied to the population of Korean older adults. Additionally, these results support the results of several studies conducted on older adults in Korea who exercise [41,42]. The finding that health beliefs influence health promotion behavior through the mediation of exercise adherence intention increases the explanatory power of the process through which older adults’ health beliefs promote their health promotion behavior. Thus, older adults’ beliefs and intentions must be bolstered to increase their health behavior, that is, exercise participation. It is important to provide diverse information about health (such as the importance of exercise, its benefits, and its drawbacks) to promote their beliefs [20]. For example, private and public sports institutions should display posters or infographics on bulletin boards explaining how regular exercise participation can positively impact health management. They should also provide information on common health issues faced by older adults and strategies to prevent and mitigate these issues. Furthermore, regular education and health information should be provided via seminars to promote health beliefs and encourage exercise continuation among older adults who participate in recreational sports.

Finally, the results of this study show that these efforts can ultimately promote successful aging in older adults. As stated earlier, successful aging benefits not only the individual but also society [9]. Considering that Korea is aging faster than other countries, successful aging is particularly essential for Korean society. However, owing to the rapid increase in the older adult population, there are many shortcomings in Korea’s public welfare system, which hinder older adults from aging successfully [42,43]. This is corroborated by the fact that, among older adults in the Organization for Economic Cooperation and Development countries, depression is the highest among Korean older adults [44]. Therefore, more research should be conducted on the successful aging of Korean older adults. There is also a need to consider the concept of truly successful aging in Korean society. With these efforts, Korean society may be able to overcome the problems faced by an aging society.

## 5. Limitations and Future Direction

Although this study provides a comprehensive understanding of health beliefs, exercise adherence intention, health promotion behavior, and successful aging among Korean older adults, it has limitations. First, because this study aimed to verify a theoretical structure, it did not analyze the individual role of the subfactors of health beliefs. The four factors that constitute health beliefs (perceived susceptibility, perceived severity, perceived benefits, and perceived obstacles) play different roles in the motivation, intention, and actual behavior of exercise participants. Therefore, future research should analyze the individual role of these subfactors based on the model of this study. Additionally, since this study targeted only those older adults who exercised regularly, it analyzed the intention to continue exercising rather than the intention to participate in exercise. Therefore, it is difficult to generalize the results of this study to older adults who wish to start exercising. Thus, future research should target older adults who want to start exercising. A comparative analysis can be performed between older adults who play a new sport and those who play the same sport. In addition, future research can expand and generalize the results of this study by adding different control variables to the model of this study. For example, by using a multi-group analysis, it will be possible to check whether the research model applies to both men and women and whether there are differences depending on the type of exercise. Finally, individual characteristics of the participants (such as sex, type of exercise, and whether one lives in an urban or rural area) were not included in the analysis. Thus, future studies should use these variables to provide more specific information. These attempts can contribute to Korean society and expand the research on the successful aging of Korean older adults who regularly engage in sports activities.

## 6. Conclusions

This study provides evidence of the structural relationships among health beliefs, exercise adherence intention, health promotion behavior, and successful aging among Korean older adults who exercise regularly. It shows that Korean older adults’ health beliefs positively predict their exercise adherence intention, health promotion behavior, and successful aging. It also shows that the HBM is a sufficiently persuasive theory, even when it is applied to Korean older adults. The model of this study can be expanded and generalized through future research and can be used to improve the health and quality of life of Korean older adults. It is also expected to help to develop policies that encourage health promotion behavior among older individuals.

## Figures and Tables

**Table 1:** Characteristics of the participants (*n* = 287).

Category		Frequency	Percentage
Sex	Male	180	62.72
	Female	107	37.28
Type of income	Earned income	143	49.83
	Pension	62	21.60
	Government subsidies	58	20.21
	Family support	24	8.36
Living situation	Living with spouse	170	59.23
	Living alone	53	18.47
	Living with children	32	11.15
	Living with family members	30	10.45
	Living with grandchildren	2	0.70
Sport	Hiking	53	18.47
	Park golf	36	12.54
	Swimming	34	11.85
	Badminton	26	9.06
	Gate ball	22	7.67
	Golf	22	7.67
	Dance	21	7.32
	Cycle	18	6.27
	Futsal	16	5.57
	Tennis	15	5.23
	Soccer	13	4.53
	Table tennis	11	3.83

**Table 2:** Results of analyzing the validity and reliability of the measurement tool of each variable.

Variable	Factors	Items	B	*β*	Standard Error	*t*	Cronbach’s α
Health beliefs	Psychological benefits	Psy 1	1.000	0.797	-	-	0.880
		Psy 2	1.060	0.866	0.067	15.937 ***	
		Psy 3	1.056	0.791	0.074	14.342 ***	
		Psy 4	0.772	0.625	0.072	10.748 ***	
		Psy 5	0.873	0.726	0.068	12.886 ***	
	Physical benefits	Phy 6	1.000	0.686	-	-	0.816
		Phy 7	1.129	0.761	0.103	10.994 ***	
		Phy 8	1.120	0.730	0.105	10.636 ***	
		Phy 9	0.990	0.730	0.093	10.634 ***	
	Social benefits	Soc 10	1.000	0.719	-	-	0.809
		Soc 11	0.970	0.722	0.092	10.508 ***	
		Soc 12	0.993	0.679	0.099	9.987 ***	
		Soc 13	1.115	0.750	0.103	10.803 ***	
	Perceived barriers	Bar 14	1.000	0.554	-	-	0.860
		Bar 15	0.714	0.321	0.161	4.447 ***	
		Bar 16	1.186	0.568	0.170	6.977 ***	
		Bar 17	1.694	0.774	0.208	8.131 ***	
		Bar 18	1.697	0.733	0.211	8.023 ***	
	Susceptibility	Sus 19	1.000	0.671	-	-	0.922
		Sus 20	0.776	0.583	0.094	8.274 ***	
		Sus 21	1.011	0.730	0.103	9.844 ***	
		Sus 22	0.971	0.663	0.106	9.181 ***	
	Severity	Sev 24	1.000	0.660	-	-	0.868
		Sev 25	1.107	0.790	0.103	10.792 ***	
		Sev 26	1.266	0.834	0.114	11.065 ***	
Exercise adherence intention		EAI 1	1.000	0.687	-	-	0.894
		EAI 2	1.170	0.781	0.079	14.714 ***	
		EAI 3	1.461	0.834	0.119	12.290 ***	
		EAI 4	1.236	0.726	0.113	10.957 ***	
		EAI 5	1.316	0.756	0.116	11.354 ***	
		EAI 6	1.249	0.772	0.108	11.551 ***	
Health promotion behavior	Health responsibility	HR 1	1.000	0.682	-	-	0.810
		HR 2	1.093	0.662	0.122	8.974 ***	
		HR 3	1.272	0.714	0.136	9.334 ***	
		HR 4	1.249	0.613	0.151	8.289 ***	
		HR 5	1.016	0.594	0.124	8.197 ***	
	Interpersonal support	IS 6	1.000	0.680	-	-	0.830
		IS 7	1.139	0.766	0.102	11.191 ***	
		IS 8	1.173	0.795	0.102	11.503 ***	
		IS 9	0.956	0.629	0.101	9.435 ***	
		IS 10	0.995	0.632	0.105	9.483 ***	
	Exercise	Exc 11	1.000	0.704	-	-	0.886
		Exc 12	1.336	0.877	0.096	13.868 ***	
		Exc 13	1.226	0.857	0.090	13.598 ***	
		Exc 14	1.063	0.748	0.089	11.961 ***	
		Exc 15	1.039	0.720	0.090	11.514 ***	
Successful aging	Autonomous life	Aut 1	1.000	0.472	-	-	0.807
		Aut 2	1.301	0.534	0.168	7.738 ***	
		Aut 3	1.258	0.512	0.203	6.183 ***	
		Aut 4	1.463	0.665	0.205	7.134 ***	
		Aut 5	1.046	0.462	0.180	5.795 ***	
		Aut 6	1.312	0.578	0.198	6.623 ***	
		Aut 7	1.379	0.510	0.223	6.195 ***	
		Aut 8	1.687	0.669	0.237	7.104 ***	
		Aut 9	1.678	0.655	0.239	7.032 ***	
	Self-completion orientation	SCO 10	1.000	0.615	-	-	0.821
		SCO 11	1.164	0.770	0.114	10.182 ***	
		SCO 12	1.067	0.714	0.110	9.665 ***	
		SCO 13	1.074	0.752	0.107	10.017 ***	
		SCO 14	1.041	0.644	0.116	8.983 ***	
	Positive life participation	PLP 16	1.000	0.827	-	-	0.912
		PLP 17	1.045	0.867	0.059	17.785 ***	
		PLP 18	1.028	0.830	0.062	16.636 ***	
		PLP 19	0.897	0.763	0.061	14.638 ***	
		PLP 20	0.870	0.791	0.056	15.428 ***	
	Satisfaction with one’s offspring	SWO 21	1.000	0.727	-	-	0.886
		SWO 22	1.467	0.845	0.105	13.932 ***	
		SWO 23	1.280	0.817	0.094	13.675 ***	
		SWO 24	1.195	0.742	0.099	12.106 ***	
		SWO 25	1.221	0.841	0.086	14.138 ***	
	Self-acceptance	SA 26	1.000	0.800	-	-	0.840
		SA 27	1.080	0.803	0.076	14.179 ***	
		SA 28	1.007	0.809	0.070	14.339 ***	
	Acceptance of others	OA 29	1.000	0.682	-	-	0.822
		OA 30	1.295	0.855	0.109	11.919 ***	
		OA 31	1.274	0.791	0.110	11.532 ***	

*** *p* < 0.001; assessed through a confirmatory factor analysis.

**Table 3:** Mean, standard deviation, skewness, kurtosis, and bivariate correlation of all subfactors.

Factor	1	2	3	4	5	6	7	8	9	10	11	12	13	14
1	-													
2	0.333 **	-												
3	0.196 **	0.041	-											
4	0.128 *	0.172 **	0.505 **	-										
5	0.256 **	0.224 **	0.118 *	0.217 **	-									
6	0.223 **	0.101	0.154 **	0.139 *	0.385 **	-								
7	0.454 **	0.405 **	0.128 *	0.139 *	0.427 **	0.379 **	-							
8	0.439 **	0.348 **	0.200 **	0.236 **	0.593 **	0.495 **	0.623 **	-						
9	0.453 **	0.409 **	0.068	0.118 *	0.200 **	0.224 **	0.566 **	0.481 **	-					
10	0.504 **	0.423 **	0.155 **	0.176 **	0.265 **	0.283 **	0.462 **	0.467 **	0.585 **	-				
11	0.449 **	0.390 **	0.144 *	0.191 **	0.219 **	0.259 **	0.585 **	0.458 **	0.634 **	0.549 **	-			
12	0.347 **	0.435 **	0.090	0.060	0.090	0.061	0.436 **	0.274 **	0.543 **	0.483 **	0.513 **	-		
13	0.389 **	0.320 **	0.027	0.040	0.115	0.125 *	0.465 **	0.295 **	0.574 **	0.490 **	0.549 **	0.609 **	-	
14	0.301 **	0.292 **	0.109	0.082	0.130 *	0.098	0.419 **	0.353 **	0.442 **	0.471 **	0.456 **	0.490 **	0.537 **	-
Mean	4.337	4.118	3.642	3.602	4.204	4.130	4.049	4.136	4.153	4.024	4.337	4.268	3.964	4.120
Standard deviation	0.438	0.592	0.705	0.754	0.723	0.552	0.656	0.457	0.567	0.658	0.558	0.608	0.743	0.648
Skewness	−0.685	−0.478	−0.186	−0.123	−1.291	−1.095	−1.164	0.114	−0.345	−0.525	−0.391	−0.340	−0.256	−1.581
Kurtosis	1.921	−0.191	−0.518	−0.650	3.205	3.181	3.679	−0.097	1.093	1.413	0.081	−0.609	−0.376	4.917

* *p* < 0.05, ** *p* < 0.01; assessed through Pearson’s correlation. Note. 1: Psychological, physical, and social benefits; 2: Perceived barriers; 3: Susceptibility; 4: Severity; 5: Exercise adherence intention; 6: Health responsibility; 7: Interpersonal support; 8: Exercise; 9: Autonomous life; 10: Self-completion orientation; 11: Positive life participation; 12: Satisfaction with one’s offspring; 13: Self-acceptance; 14: Acceptance of others.

**Table 4:** Results of verifying the measurement model.

Variables	Factors	B	*β*	Standard Error	*t*
Health beliefs	Susceptibility	1.000	0.573	-	-
	Severity	1.368	0.548	0.397	3.748 ***
	Perceived barriers	1.464	0.509	0.401	3.648 ***
	Benefits	1.345	0.632	0.355	3.795 ***
Exercise adherence intention	Parceling 1	1.000	0.853	-	-
	Parceling 2	0.948	0.868	0.056	16.797 ***
	Parceling 3	0.881	0.783	0.059	14.988 ***
Health promotion behavior	Health responsibility	1.000	0.533	-	-
	Interpersonal support	1.113	0.722	0.134	8.338 ***
	Exercise	1.602	0.873	0.179	8.950 ***
Successful aging	Autonomous life	1.000	0.795	-	-
	Self-completion orientation	1.110	0.713	0.089	12.475 ***
	Positive life participation	1.403	0.775	0.102	13.798 ***
	Satisfaction with one’s offspring	1.074	0.700	0.088	12.218 ***
	Self-acceptance	1.232	0.737	0.095	12.980 ***
	Acceptance of others	1.290	0.631	0.119	10.826 ***

*** *p* < 0.001; assessed through a confirmatory factor analysis.

**Table 5:** Estimate and standardized estimate of the direct paths.

Direct Path	Estimate	Standard Estimate	Standard Error	*t*
Health beliefs → Exercise adherence intention	1.476	0.473	0.455	3.242 ***
Exercise adherence intention → Health promotion behavior	0.204	0.366	0.050	4.095 ***
Health beliefs → Health promotion behavior	1.201	0.691	0.363	3.305 ***
Health promotion behavior → Successful aging	0.641	0.631	0.091	7.036 ***

*** *p* < 0.001; assessed through a structural equation model analysis.

**Table 6:** Estimate of the indirect paths.

Indirect Path	Estimate	Standard Error	95% Confidence Interval	
			Lower	Upper
Health beliefs → Exercise adherence intention → Health promotion behavior	0.301 *	0.206	0.079	1.156
Health beliefs → Health promotion behavior → Successful aging	0.770 **	0.528	0.324	1.941
Health beliefs → Exercise adherence intention → Health promotion behavior → Successful aging	0.193 **	0.129	0.073	0.914

* *p* < 0.05, ** *p* < 0.01; assessed through a structural equation model analysis.

## Data Availability

The data supporting the findings of this study are available from the corresponding author upon request.
